# Supplementary material for: Optical Tellegen metamaterial with spontaneous magnetization
Source: Nat Commun. 2024 Feb 12;15:1293. doi: 10.1038/s41467-024-45225-y (PMC10861567; doi:10.1038/s41467-024-45225-y)
Supplement: Supplementary file 1 — Supplementary Information [file 41467_2024_45225_MOESM1_ESM.pdf]

Supplementary material for the paper

"Optical Tellegen metamaterial with spontaneous magnetization"

Shadi Safaei Jazi, Ihar Faniayeu, Rafael Cichelero, Dimitrios C. Tzarouchis, Mohammad Mahdi Asgari, Alexandre Dmitriev, Shanhui Fan, and Viktar Asadchy

Contents

1 Finding the largest dimensions of a single-domain cylindrical magnetic particle 2

2 Finding the smallest dimensions of a single-domain cylindrical magnetic particle 3

3 Coercive and stray fields of a single-domain cylindrical magnetic particle 5

4 Symmetry constraints on the polarizability tensors of the meta-atom 7

5 Polarizability components of the cobalt-based Tellegen meta-atom and the effective-medium theory 9

6 Dielectric tensor of the magnetic Weyl semimetal 10

7 Polarizability components of the Weyl-based Tellegen meta-atom 11

1 Finding the largest dimensions of a single-domain cylindrical magnetic particle

In this derivation, we assume that the owest-energy configuration of the domain wall is along the cylinder axis dividing it into two halves with opposite magnetization orientations, as shown in Supplementary Figure 1. We also assume that the aspect ratio of the ferromagnetic cylinder is of the order of unit $h_{\mathrm{Co}}/D = \delta \sim 1$. The total energy of a cylinder in the single-domain configuration (see Supplementary Figure 1a) is given by the magnetostatic energy $\mathcal{E}_{\mathrm{ms,I}} = \frac{N_{\mathrm{c}}}{2}\mu_0 M_{\mathrm{s}}^2 V$, where $V = \pi\delta D^3/4$ is the cylinder volume and

$$N_{\mathrm{c}} = 1 - \delta/\sqrt{1+\delta^2} \qquad \text{(S1)}$$

is the demagnetizing factor [S1, p. 37].

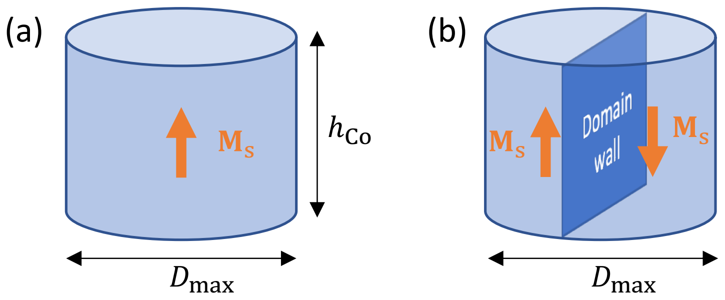

Supplementary Figure 1: Energy balance in the ferromagnetic nanocylinder. For the diameter $D = D_{\mathrm{max}}$, the single-domain (a) and two-domain (b) configurations have equal potential energies.

The total energy of a cylinder in the two-domain configuration (see Supplementary Figure 1b equals the sum of the magnetostatic energy (which is now approximately twice smaller, that is, $\mathcal{E}_{\mathrm{ms,II}} \approx \mathcal{E}_{\mathrm{ms,I}}/2$) and the domain wall energy $\mathcal{E}_{\mathrm{w}} = \gamma\delta D^2$ [S2, Sec. 17.5]. Here, γ is the total surface energy of the wall given by $\gamma = 4\sqrt{AK_1}$ with K_1 being the magnetocrystalline anisotropy constant and A is the exchange stiffness coefficient ($K_1 = 410$ kJ m^{-3} and $A = 31$ pJ m^{-1} for cobalt [S1]).

In order to find the maximal diameter D_{max} (for a given δ) of a single-domain cylinder,

we consider the energy balance $\mathcal{E}_{\mathrm{ms,I}} = \mathcal{E}_{\mathrm{ms,II}} + \mathcal{E}_{\mathrm{w}}$. After simplification, we obtain

$$D_{\mathrm{max}} = \frac{64\sqrt{AK_1}}{\pi N_{\mathrm{c}} \mu_0 M_{\mathrm{s}}^2}. \tag{S2}$$

Supplementary Figure 2 plots the maximal diameter versus the aspect ratio for a cobalt nanocylinder.

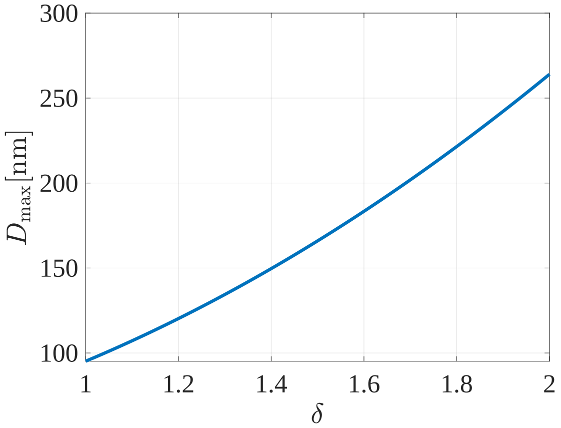

Supplementary Figure 2: Maximal diameter of a single-domain cobalt cylinder as a function of its aspect ratio δ.

2 Finding the smallest dimensions of a single-domain cylindrical magnetic particle

The single-domain magnetic state with a fixed magnetization direction can be enforced by various types of anisotropies, including magnetocrystalline and shape anisotropies. In this case, in order to tilt magnetization in the nanocylinder away from the anisotropy axis by an angle θ, one needs to apply the amount of energy equal to $\mathcal{E}_{\mathrm{a}} = (K_1 + K_{\mathrm{sh}})V \sin^2\theta$, where V is the cylinder volume, K_1 is the magnetocrystalline anisotropy coefficient, and $K_{\mathrm{sh}} = \frac{\mu_0}{4} M_{\mathrm{s}}^2 (1 - 3N_{\mathrm{c}})$ is the shape anisotropy coefficient (here, we assume that both anisotropies have the same axis). To flip the magnetization direction to the opposite one,

it is sufficient to tilt it at the angle $\theta = \pi/2$. Thus, the minimum energy for the flip is $\mathcal{E}_{\rm a} = (K_1 + K_{\rm sh})V$.

When the volume of the ferromagnetic cylinder becomes too small, the anisotropy energy $\mathcal{E}_{\rm a}$ can become comparable in value to the thermal energy $k_{\rm B}T$. In such circumstances, the cylinder constitutes a single domain state but its magnetization randomly flips orientation. This superparamagnetic state is undesirable for our design of the Tellegen meta-atom. Let us estimate the minimal cylinder diameter at which the transition from ferromagnetic to superparamagnetic state occurs.

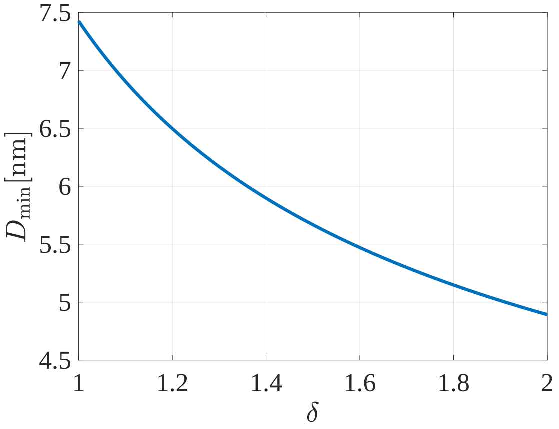

Supplementary Figure 3: Minimal diameter of a single-domain cobalt cylinder as a function of its aspect ratio δ.

Due to the stochastic nature of thermal energy, magnetization flip is a time-dependent process that can be described by the following probability distribution [S3]

$$\tau_{\rm flip} = \tau_{\rm ch} {\rm e}^{-\mathcal{E}_{\rm a}/k_{\rm B}T}. \tag{S3}$$

Here, $\tau_{\rm ch}$ stands for the characteristic time scale over which electron spins attempt to jump the barrier energy $\mathcal{E}_{\rm a}$ and $\tau_{\rm flip}$ is the average time required for the magnetization to flip in the cylinder at temperature T. From here, one can derive the minimum diameter

of the cobalt cylinder in the single-domain ferromagnetic state:

$$D_{\mathrm{min}} = \sqrt[3]{\frac{4k_{\mathrm{B}}T}{\pi\delta(K_1 + K_{\mathrm{sh}})} \ln\left(\tau_{\mathrm{flip}}/\tau_{\mathrm{ch}}\right)}, \tag{S4}$$

where ln denotes the natural logarithm. Choosing $\tau_{\mathrm{flip}} \sim 1$ year (to ensure repeatable experimental measurements), $\tau_{\mathrm{ch}} \sim 1$ ns, and room temperature $T = 300$ K, the minimal diameter as a function of the aspect ratio of the cobalt cylinder is plotted in Supplementary Figure 3.

3 Coercive and stray fields of a single-domain cylindrical magnetic particle

Let us find the coercive field of the ferromagnetic cylinder. The coercive field is the minimal magnetic field that needs to be applied to the cylinder in the direction opposite to its magnetization so that it flips the magnetization. Ideally, this field must be sufficiently large so that the meta-atom is not demagnetized when it is located in the random mixture of equivalent meta-atoms (by their stray fields). Assume that an external static magnetic field $\mathbf{H}$ is applied to the cylinder as depicted in Supplementary Figure 4. Let $\mathbf{H}$ and

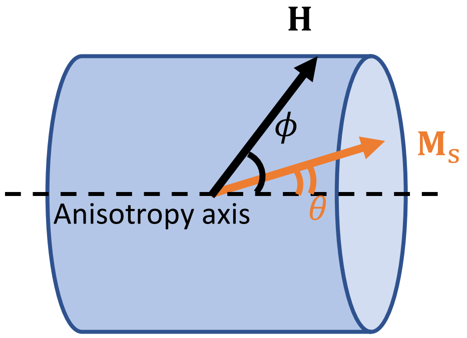

Supplementary Figure 4: Geometry of the problem for the calculation of the coercive field in the ferromagnetic cylinder.

$\mathbf{M}_{\mathrm{s}}$ to form angles ϕ and θ with respect to the anisotropy axis, respectively. The total

potential energy of the cylinder is given by [S1, Sec. 7.3.1]

$$\mathcal{E}_{\text{tot}}(\theta) = (K_1 + K_{\text{sh}}) \sin^2 \theta - M_{\text{s}} H \cos(\phi - \theta)\mu_0, \tag{S5}$$

where the first term is the energy due to the magnetic anisotropy and the second term is due to the external field. By choosing $\phi = \pi$ and requiring $\frac{\mathrm{d}^2}{d\theta^2}\mathcal{E}_{\text{tot}}(0) = 0$ (the state with original magnetization reaches an unstable energy minimum), we find the coercive field

$$H_{\text{c}} = \frac{2(K_1 + K_{\text{sh}})}{\mu_0 M_{\text{s}}}, \tag{S6}$$

Supplementary Figure 5a plots the coercive field of a single-domain cobalt cylinder versus its aspect ratio.

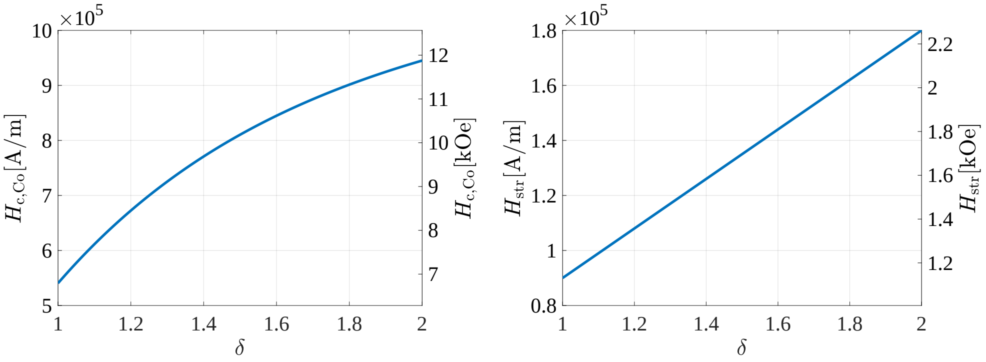

(a) (b)

Supplementary Figure 5: (a) Coercive field and (b) stray field of the cobalt cylinder for different aspect ratio values.

The stray field produced by a single-domain cylinder at a distance R from its axis can be found as [S1, Eq. 2.10]

$$H_{\text{str}} = \frac{M_{\text{s}} \delta D^3}{16 R^3}. \tag{S7}$$

This expression holds in the assumption that the cylinder can be modeled as a magnetic point dipole. Supplementary Figure 5b plots the stray field for the case when $R = D$,

which corresponds to a very compact meta-atom packing in a mixture. As one can see, even in for such dense packing, the stray field is multiple times weaker than the coercive field of the magnetic meta-atoms. This circumstance means that the meta-atoms possess stable magnetization and cannot demagnetize one another when mixed randomly in a composite.

4 Symmetry constraints on the polarizability tensors of the meta-atom

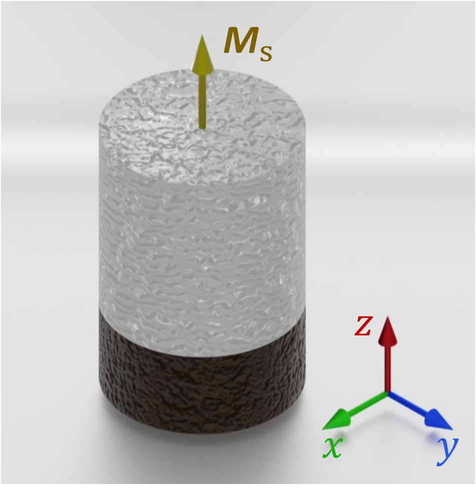

Supplementary Figure 6: Considered meta-atom comprises a ferromagnetic nanocylinder in the single-domain state (upper part) and a high-index nanocylinder (lower part) in the chosen coordinate basis.

The considered meta-atom shown in Supplementary Figure 6 includes only the following symmetry operations: $\underline{m}_x$, $\underline{m}_y$, and 4_z. Here, the 4_z operator denotes a four-fold rotational symmetry with a three-dimensional matrix representation given by

$$\overline{\overline{R}}_{4z} = \begin{pmatrix} 0 & -1 & 0 \\ 1 & 0 & 0 \\ 0 & 0 & 1 \end{pmatrix}.$$

Here, the double bar denotes a tensor quantity. The matrices representing the mirror

symmetry operators m_x and m_y (without time reversal application) are given by

$$\overline{\overline{m}}_x = \begin{pmatrix} -1 & 0 & 0 \\ 0 & 1 & 0 \\ 0 & 0 & 1 \end{pmatrix}, \qquad \overline{\overline{m}}_y = \begin{pmatrix} 1 & 0 & 0 \\ 0 & -1 & 0 \\ 0 & 0 & 1 \end{pmatrix}.$$

If a meta-atom has a group of symmetry, its material tensors are invariant under the operators of this group. Let us assume first the meta-atom with the most general form of polarizability tensors:

$$\mathbf{p} = \overline{\overline{\alpha}}_{\rm ee}\mathbf{E} + \overline{\overline{\alpha}}_{\rm em}\mathbf{H}, \qquad \mathbf{m} = \overline{\overline{\alpha}}_{\rm me}\mathbf{E} + \overline{\overline{\alpha}}_{\rm mm}\mathbf{H}, \tag{S8}$$

In what follows, considering all the generators of the symmetry group of the meta-atom, we can find the constrained form of its polarizability tensors.

For the 4_z rotational operator, the following symmetry relations must hold [S4]:

$$\overline{\overline{R}}_{4z}\,\overline{\overline{\alpha}}_{\rm ee} = \overline{\overline{\alpha}}_{\rm ee}\,\overline{\overline{R}}_{4z}, \qquad \overline{\overline{R}}_{4z}\,\overline{\overline{\alpha}}_{\rm mm} = \overline{\overline{\alpha}}_{\rm mm}\,\overline{\overline{R}}_{4z}, \tag{S9}$$

$$\overline{\overline{R}}_{4z}\,\overline{\overline{\alpha}}_{\rm em} = \det\{\overline{\overline{R}}_{4z}\}\,\overline{\overline{\alpha}}_{\rm em}\,\overline{\overline{R}}_{4z}, \qquad \overline{\overline{R}}_{4z}\,\overline{\overline{\alpha}}_{\rm me} = \det\{\overline{\overline{R}}_{4z}\}\,\overline{\overline{\alpha}}_{\rm me}\,\overline{\overline{R}}_{4z}. \tag{S10}$$

Note that in Supplementary Equation(S10), the determinant of matrix $\overline{\overline{R}}_{4z}$ appears due to the fact that $\overline{\overline{\alpha}}_{\rm em}$ and $\overline{\overline{\alpha}}_{\rm me}$ are pseudotensors (axial tensors) and transform differently compared to polar tensors, such as $\overline{\overline{\alpha}}_{\rm ee}$ and $\overline{\overline{\alpha}}_{\rm mm}$ [S4].

For the antiunitary operators $\underline{m}_x$ (which is a product of a respective usual geometrical symmetry operator and the time-reversal operator), the symmetry relations read

$$\overline{\overline{m}}_x\,\overline{\overline{\alpha}}_{\rm ee} = \overline{\overline{\alpha}}_{\rm ee}^T\,\overline{\overline{m}}_x, \qquad \overline{\overline{m}}_x\,\overline{\overline{\alpha}}_{\rm mm} = \overline{\overline{\alpha}}_{\rm mm}^T\,\overline{\overline{m}}_x, \tag{S11}$$

$$\overline{\overline{m}}_x\,\overline{\overline{\alpha}}_{\rm em} = -\det\{\overline{\overline{m}}_x\}\,\overline{\overline{\alpha}}_{\rm me}^T\,\overline{\overline{m}}_x, \qquad \overline{\overline{m}}_x\,\overline{\overline{\alpha}}_{\rm me} = -\det\{\overline{\overline{m}}_x\}\,\overline{\overline{\alpha}}_{\rm em}^T\,\overline{\overline{m}}_x, \tag{S12}$$

where T is the transpose matrix operator. Similar relations hold also for $\underline{\underline{m}}_y$ operator.

By combining Supplementary Equations (S9)–(S12), we find that due to the symmetry of the meta-atom, its polarizability tensors must have the following forms:

$$\overline{\overline{\alpha}}_{\rm ee} = \begin{pmatrix} \alpha_{\rm ee}^{xx} & \alpha_{\rm ee}^{xy} & 0 \\ -\alpha_{\rm ee}^{xy} & \alpha_{\rm ee}^{xx} & 0 \\ 0 & 0 & \alpha_{\rm ee}^{zz} \end{pmatrix}, \qquad \overline{\overline{\alpha}}_{\rm mm} = \begin{pmatrix} \alpha_{\rm mm}^{xx} & \alpha_{\rm mm}^{xy} & 0 \\ -\alpha_{\rm mm}^{xy} & \alpha_{\rm mm}^{xx} & 0 \\ 0 & 0 & \alpha_{mm}^{zz} \end{pmatrix}, \tag{S13}$$

$$\overline{\overline{\alpha}}_{\rm em} = \begin{pmatrix} \alpha_{\rm em}^{xx} & \alpha_{\rm em}^{xy} & 0 \\ -\alpha_{\rm em}^{xy} & \alpha_{\rm em}^{xx} & 0 \\ 0 & 0 & \alpha_{\rm em}^{zz} \end{pmatrix}, \qquad \overline{\overline{\alpha}}_{\rm me} = \begin{pmatrix} \alpha_{\rm em}^{xx} & \alpha_{\rm em}^{xy} & 0 \\ -\alpha_{\rm em}^{xy} & \alpha_{\rm em}^{xx} & 0 \\ 0 & 0 & \alpha_{\rm em}^{zz} \end{pmatrix}. \tag{S14}$$

Using the Onsager-Casimir relations [S5], we can write the following relations from Supplementary Equations (S13)–(S14) in the notations used in the main text:

$$\alpha_{\rm e} = \alpha_{\rm ee}^{xx} \neq 0, \qquad \alpha_{\rm m} = \alpha_{\rm mm}^{xx} \neq 0, \qquad \alpha_{\rm ge} = -\alpha_{\rm ee}^{xy} \neq 0, \qquad \alpha_{\rm gm} = -\alpha_{\rm mm}^{xy} \neq 0, \tag{S15}$$

$$\alpha_{\chi{\rm s}} = \alpha_{\rm em}^{xx} \neq 0, \qquad \alpha_{\kappa{\rm a}} = -\alpha_{\rm em}^{xy} \neq 0, \qquad \alpha_{\chi{\rm a}} = 0, \qquad \alpha_{\kappa{\rm s}} = 0. \tag{S16}$$

Thus, one can see that chirality, described by $\alpha_{\kappa{\rm s}}$, is forbidden in the considered meta-atom by symmetry.

5 Polarizability components of the cobalt-based Tellegen meta-atom and the effective-medium theory

Supplementary Figure 7 plots the six extracted diagonal polarizability components that solely contribute to the bulk isotropic response in random mixtures of meta-atoms shown in Fig. 2a and 2b in the main text. The polarizabilities are normalized to the same units using the vacuum wave impedance $\eta_0 = \mu_0 c$. Note that $\alpha_{\rm e}$, $\alpha_{\rm m}$, and $\alpha_{\chi{\rm s}}$ stand for the in-plane (xx and yy) components. The first observation one can make from the figure is

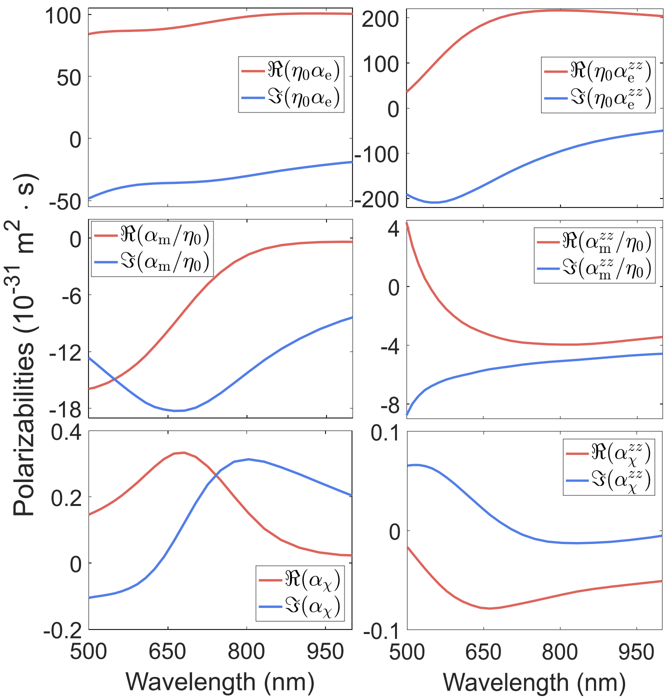

Supplementary Figure 7: Normalized polarizability components of the proposed cobalt-silicon meta-atom. The real and imaginary parts are $\Re$ and $\Im$ operators, respectively.

that although the electric polarizability components are dominant, the meta-atom indeed exhibits a magnetic Mie-type resonance (as was required by our design) expressed by the Lorentzian-like behavior of $\alpha_{\rm m}$ component. As a consequence, the meta-atom has the resonant enhancement of the magnetoelectric polarizabilities $\alpha_{\chi{\rm s}}$ and $\alpha_{\chi{\rm s}}^{zz}$. The resonance for the former one occurs near the 750 nm wavelength.

6 Dielectric tensor of the magnetic Weyl semimetal

For the sake of self-sufficiency, we plot in Supplementary Figure 8 the frequency dispersion of the diagonal $\varepsilon_{xx} = \varepsilon_{yy}$ and off-diagonal $\varepsilon_{xy} = -\varepsilon_{yx}$ permittivity components of bulk Weyl semimetal $EuCd_2As_2$ with the materials parameters defined as in [S6]. The semimetal magnetization is along the z-axis.

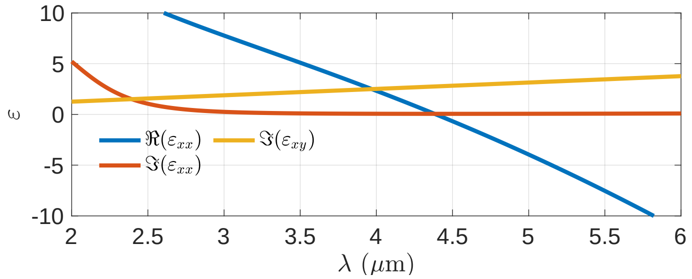

Supplementary Figure 8: Frequency dispersion of the permittivity components of magnetic Weyl semimetal $EuCd_2As_2$.

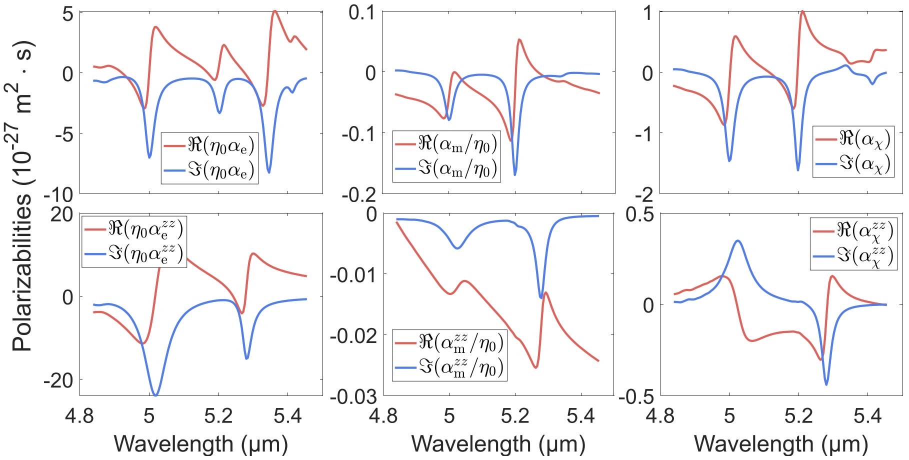

Supplementary Figure 9: Normalized polarizability components of the Weyl-based Tellegen meta-atom shown in Figure 3a in the main text. The real and imaginary parts are shown as $\Re$ and $\Im$ operators, respectively.

7 Polarizability components of the Weyl-based Tellegen meta-atom

In Supplementary Figure 9, we plot the six independent polarizability components of Weyl-based meta-atom shown in Figure 3a of the main text that solely contribute to the bulk isotropic response in random mixtures of such meta-atoms.

References

[S1] J. M. D. Coey. *Magnetism and Magnetic Materials.* Cambridge University Press, March 2010.

[S2] Soshin Chikazumi. *Physics of Ferromagnetism*. OUP Oxford, April 2009.

[S3] C. M. Sorensen. Magnetism. In *Nanoscale Materials in Chemistry*, chapter 6, pages 169–221. John Wiley & Sons, Ltd, 2001.

[S4] V. Dmitriev. Group theoretical approach to complex and bianisotropic media description. *The European Physical Journal - Applied Physics*, 6(1):49–55, April 1999.

[S5] V. S. Asadchy, M. S. Mirmoosa, A. Díaz-Rubio, S. Fan, and S. A. Tretyakov. Tutorial on electromagnetic nonreciprocity and its origins. *Proceedings of the IEEE*, 108(10):1684–1727, October 2020.

[S6] Viktar S. Asadchy, Cheng Guo, Bo Zhao, and Shanhui Fan. Sub-wavelength passive optical isolators using photonic structures based on weyl semimetals. *Advanced Optical Materials*, 8(16):2000100, 2020.
